# Bio-Inspired Adaptive Multimodal Decision Fusion for Intelligent Safety Monitoring in Confined Spaces

**DOI:** 10.3390/biomimetics11060367

**Published:** 2026-05-25

**Authors:** Xinhai Li, Zhibin Lian, Heng Zhou, Qiang Zhou

**Affiliations:** 1 The Zhongshan Power Supply Bureau, Guangdong Power Grid Co., Ltd., Zhongshan 528400, China; 2 School of Electrical and Electronic Engineering, Shandong University of Technology, Zibo 255049, China

**Keywords:** intelligent safety monitoring, human activity recognition, multimodal decision fusion, LSTM, FFT, bio-inspired computing, adaptive inverse effectiveness

## Abstract

To improve operational safety in confined spaces, this study proposes an intelligent safety monitoring framework that utilizes multimodal data from wearable devices. The framework comprises two core components: a human activity recognition (HAR) module and a bio-inspired adaptive multimodal decision fusion (BA-MDF) module. The HAR module processes accelerometer and gyroscope data through an enhanced FFT–LSTM architecture that integrates time- and frequency-domain features for real-time activity classification. The BA-MDF module, inspired by biological multisensory integration mechanisms—particularly the inverse effectiveness principle observed in the superior colliculus—evaluates contextual risk by adaptively fusing HAR outputs, heart rate variability, and geospatial constraints without additional computational overhead. Experimental testing demonstrated 92.4% overall HAR accuracy and 94.3% identification accuracy for emergency scenarios under a simulated sensor degradation environment. These results validate the framework’s effectiveness in mitigating risks from anomalous events in visually constrained environments.

## 1. Introduction

With the development of information and communication technologies (ICT), intelligent safety monitoring methods have attracted global attention in recent years, particularly within high-risk confined environments such as underground mines, industrial tanks, and confined construction zones [1]. While existing ICT-based approaches have demonstrated their ability to detect potential anomalous situations, most of them rely on visual surveillance systems [1,2]. However, such vision-dependent approaches face critical limitations in visually degraded conditions—such as fog, smoke, dust, or low-light scenarios—commonly encountered in industrial settings. These limitations underscore the need for alternative strategies to reliably detect anomalous situations in visually degraded cases.

Human activity recognition (HAR) serves as the fundamental step in intelligent safety monitoring. Extensive research efforts have focused on HAR methodologies that fuse accelerometer and gyroscope measurements [3]. The strength of these methodologies lies in their capability to classify activities within visual surveillance blind zones, where traditional vision-based monitoring exhibits inherent limitations. Several studies have utilized wearable sensors to collect accelerometer and gyroscope data for analyzing walking patterns (e.g., level walking, ascending/descending stairs) and stationary postures (e.g., sitting, standing, lying) [4,5,6]. These studies have predominantly employed deep learning architectures, with long short-term memory (LSTM) networks serving as the primary architecture for activity classification. Parallel to this, researchers have developed fall detection methods through machine learning, including support vector machines (SVM), recurrent neural networks (RNN), and LSTM, using inertial sensor data from smartphones or smartwatches [7,8,9]. Further, technologies for detecting anomalous activities were introduced in prior research [10,11], which rely on analyzing acceleration magnitudes derived from smartwatch-based accelerometers and gyroscopes. Collectively, these LSTM-based HAR implementations demonstrate approximately 90% accuracy.

Despite these advancements, a critical limitation persists: for a recognized activity, existing approaches based on accelerometer and gyroscope data face challenges in distinguishing between normal and anomalous patterns. A particularly challenging scenario arises when distinguishing between human falls and accidental device drops, as the sensor readings captured during a person’s fall closely resemble those recorded when a smartphone is simply dropped to the ground. Consequently, existing techniques are unable to effectively discriminate between the critical event of human falling and the commonplace occurrence of device dropping. This ambiguity leads to an unacceptably high rate of false alarms, eroding system trustworthiness in life-critical applications.

The cross-analysis of multimodal data can be used to improve safety monitoring, and, to overcome this fundamental limitation, the present work puts forward a novel bio-inspired adaptive multimodal decision fusion (BA-MDF) framework that transcends single-sensor HAR by integrating three complementary data streams:

- IMU-based activity classification enhanced through attitude and heading reference system (AHRS) for orientation-aware feature extraction, followed by fast Fourier transform (FFT) for spectral pattern recognition and LSTM for temporal sequence modeling;
- Physiological monitoring via continuous heart rate variability (HRV) from wearable smartwatches, serving as a biomarker for stress-induced physiological response;
- Spatial context derived from GPS positioning, enabling detection of group-level anomalies (e.g., multiple individuals in prolonged supine positions within a 5 m radius).

The integration of these modalities enables context-aware risk inference: a detected “lying” posture is not interpreted in isolation. Instead, it is validated through cross-modal correlation—elevated HRV indicates physiological distress, while spatial adjacency confirms potential multi-person collapse. This multi-layered inference significantly reduces false positives while enhancing sensitivity to true emergencies.

Unlike prior works treating HAR as a standalone classification task, our approach redefines activity recognition as the first layer of a risk-inference pipeline, where contextual and physiological cues serve as essential validation signals. This paper makes three key contributions:

(1) A novel AHRS–FFT–LSTM pipeline for orientation-resilient HAR in dynamic, body-mounted IMU configurations;
(2) The first fusion of HRV and GPS spatial clustering with HAR outputs for hazard discrimination in confined spaces, enabling adaptive weight adjustment based on real-time sensor reliability;
(3) A lightweight, real-time BA-MDF decision logic with adaptive risk scoring, validated under field-deployable constraints.

The remainder of this paper is structured as follows: Section 2 provides an overview of related work in HAR and safety monitoring. Section 3.1 outlines the overall system architecture. Section 3.2 details the HAR module design, including an enhanced FFT–LSTM model for activity recognition. Section 3.3 details the BA-MDF module for multimodal fusion and decision-making. Section 4.1 presents the performance evaluation of HAR using public datasets. Section 4.2 presents system validation through controlled experiments. Section 5 concludes the paper and outlines future research directions.

## 2. Related Work

Prior studies centered on intelligent safety monitoring are surveyed and critically examined in this section. This study mainly focuses on three major aspects.

### 2.1. Vision-Based Monitoring and IMU Sensing for Activity Recognition

Vision-based surveillance has long dominated intelligent safety monitoring in industrial environments. Recent advancements in deep learning have enabled robust HAR through RGB and depth sensors, with performance exceeding 90% under controlled lighting conditions [12]. However, in confined industrial environments characterized by dust, smoke, and low illumination, optical sensors suffer severe degradation due to light scattering and absorption, resulting in loss of spatial details and unreliable pose estimation [13].

To mitigate these limitations, inertial measurement units (IMUs) have emerged as a resilient alternative, leveraging accelerometer and gyroscope data to classify activities without reliance on visual input. LSTM networks have proven effective in modeling temporal dynamics of IMU sequences, sustaining classification accuracy near 90% in dynamic settings [14]. Further, FFT enables spectral feature extraction, capturing distinct frequency signatures of motion patterns [15]. Recent hybrid CNN–LSTM architectures using wrist-worn sensors have achieved a fall detection accuracy of 96.94%, demonstrating the potential of deep learning in distinguishing complex motion patterns [16]. Despite these advances, IMU-only systems remain challenged in discriminating between genuine human falls and incidental device drops, as their inertial signatures exhibit high similarity, leading to elevated false alarm rates in safety-critical contexts [14]. This inherent ambiguity underscores the necessity of multimodal validation to contextualize anomalous events.

### 2.2. Physiological Monitoring and Spatial Context Awareness

Anomalous heart rate patterns serve as physiological indicators of both physical exertion and psychological strain [17]. Heart rate variability (HRV), derived from photoplethysmography (PPG) or electrocardiogram (ECG) signals, is a well-established biomarker of autonomic nervous system activity and physiological stress. Metrics such as SDNN, RMSSD, and LF/HF ratio demonstrate strong correlations with acute stress states, with machine learning classifiers such as random forest, SVM, and deep neural networks achieving up to 99% accuracy in stress prediction [18,19] Nevertheless, HRV alone provides limited insight into comprehensive physiological status, as it primarily reflects sympathetic activation and is confounded by non-stress-related factors such as physical exertion or thermal load [20].

Location-aware safety systems have evolved from individual tracking to spatial clustering and proximity analysis for group anomaly detection. Technologies such as GPS and ultra-wideband (UWB) enable real-time worker localization, supporting emergency mustering, zone compliance, and man-down alerting [21,22]. In confined industrial environments such as underground mines and utility tunnels, UWB overcomes signal blockage issues inherent to GPS, providing reliable positioning where satellite signals are unavailable [22,23]. Industrial deployments now leverage cloud-based platforms to monitor multiple personnel simultaneously, aligning with OSHA and CSA Z1006-16 mandates for continuous surveillance and rapid response [24,25,26].

### 2.3. Multimodal Fusion Strategies

Multimodal fusion frameworks have been proposed to overcome unimodal constraints, broadly categorized into early (feature-level) and late (decision-level) fusion. Recent architectures employ multi-head CNNs with attention mechanisms to fuse visual and inertial streams, achieving classification accuracies exceeding 97% [15,27]. Decision-level fusion, particularly through logarithmic opinion pooling (LOP) and late fusion modules, provides critical advantages in safety systems by enabling weighted risk scoring and uncertainty quantification [27,28]. Studies indicate that late fusion strategies yield a 5.0% performance improvement over feature-level decision fusion for cross-view tasks while maintaining superior generalization capabilities across diverse datasets [27]. However, most existing approaches prioritize classification performance over real-time deployability, and the integration of physiological signals with spatial context for group-level anomaly detection remains underexplored.

Recent advances in confined space safety monitoring highlight the transformative potential of integrated IoT solutions. Raksha-IoT platforms combining RFID and BLE Beacon technology enable real-time, hands-free tracking of workers’ movements with health parameter monitoring including body temperature, SpO_2_, and pulse [29]. 5G-enabled manhole antenna technologies extend network coverage into underground facilities, integrating gas detection, water level monitoring, and AI-driven visual perception for comprehensive environmental and behavioral monitoring [30]. These developments underscore the critical need for multimodal systems that combine physiological, kinematic, and spatial data for robust safety assurance in high-risk industrial environments.

### 2.4. Bio-Inspired Adaptive Fusion: Inverse Effectiveness

While traditional fusion approaches employ fixed or empirically determined weights, biological systems demonstrate sophisticated adaptive multisensory integration mechanisms. The superior colliculus (SC) in the mammalian midbrain—a primary model for multisensory integration—exhibits the inverse effectiveness principle: multisensory enhancement is strongest when individual unimodal stimuli are weak [31]. Neurophysiological studies demonstrate that SC neurons show maximal cross-modal facilitation when visual or auditory inputs are near detection thresholds, with response enhancement often exceeding the sum of unimodal responses [32].

This biological principle offers a compelling framework for industrial safety monitoring in confined spaces. When visual surveillance degrades due to smoke or dust, or when GPS signals attenuate in underground environments, a bio-inspired system should automatically enhance the weighting of remaining reliable modalities (IMU, heart rate) to maintain detection performance. Unlike conventional fixed-weight fusion that suffers catastrophic performance drops under sensor degradation, inverse effectiveness provides a theoretically grounded mechanism for dynamic weight adaptation based on real-time sensory reliability.

Recent applications of inverse effectiveness to artificial systems have demonstrated improved robustness under sensor occlusion and noise [33]. For confined space safety, where environmental conditions unpredictably degrade specific sensor modalities, bio-inspired adaptive weighting offers a biologically plausible solution that does not require additional computational resources beyond reliability estimation.

## 3. Methodology

### 3.1. Overall Architecture for BA-MDF-Based Safety Monitoring

**Figure 1:**
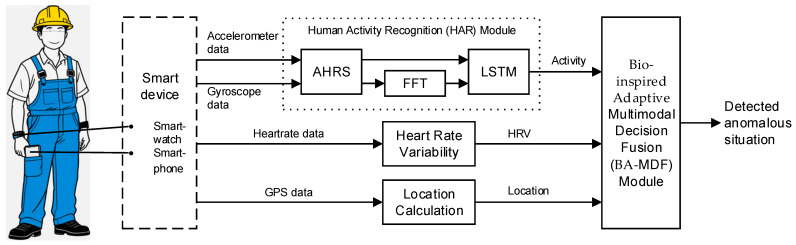
Schematic overview of the proposed safety monitoring framework.

Figure 1 illustrates the comprehensive architecture of the proposed multimodal safety monitoring system designed for real-time hazard detection among workers in confined spaces. The system integrates multiple sensor modalities—accelerometer, gyroscope, GPS, and heart rate—to enable comprehensive situational awareness. The architecture is structured into two tightly coupled modules: human activity recognition (HAR) and bio-inspired adaptive multimodal decision fusion (BA-MDF), each serving distinct yet synergistic functions.

The HAR module performs fine-grained activity classification by processing high-frequency IMU data (accelerometer and gyroscope) sampled at 50 Hz from wearable devices. Raw triaxial acceleration and angular velocity signals are preprocessed and fed into a hybrid pipeline comprising:

- Attitude and heading reference system (AHRS): reconstructs 3D device orientation in real time;
- Fast Fourier transform (FFT): extracts spectral features from time-series inertial data;
- Long short-term memory (LSTM): captures temporal dynamics to distinguish among activities including walking, sitting, and lying.

Concurrently, GPS data provides geospatial coordinates and velocity estimates, while HRV from a smartwatch serves as a physiological indicator of stress, enabling the detection of both physical exertion and cognitive load.

The BA-MDF module serves as the system’s risk inference and alerting layer, integrating HAR outputs, HRV trends, and geospatial constraints into a unified decision framework. It evaluates contextual risk using an adaptive scoring mechanism that maps activity states against predefined safety thresholds (e.g., prolonged stationary posture in restricted zones, elevated heart rate during high-risk tasks). Deviations exceeding predefined thresholds trigger tiered alarm signals via deterministic rule-based logic.

The proposed framework is designed as a centralized processing architecture. Wearable devices (e.g., smartwatches) function exclusively as sensing peripherals, responsible solely for raw data acquisition. All computationally intensive tasks—including FFT–LSTM-based HAR inference, bio-inspired adaptive fusion scoring, and anomalous state decision-making—are executed on the industrial PC.

Implementation of the HAR module is described in Section 3.2, with emphasis on model enhancement and implementation of HAR using the enhanced model. The full specification of BA-MDF, including data-fusion rules and hazard-tiered responses, is detailed in Section 3.3.

### 3.2. Human Activity Recognition (HAR) Module

#### 3.2.1. An Enhanced FFT–LSTM Model for HAR

In this section, the foundational LSTM architecture for time-series analysis is outlined, followed by a concise exposition of Fourier transformation principles. The proposed FFT–LSTM model is then formulated to synergistically integrate time- and frequency-domain features, thereby enhancing the accuracy of long-term forecasting.

A. Long Short-Term Memory Network

As a specialized variant of RNNs, LSTM effectively addresses the long-term dependency limitation commonly encountered in conventional RNN architectures when training long sequences of data [34]. Its unique gating mechanism enables information retention across long sequences, rendering LSTM highly suitable for temporal prediction tasks. The model demonstrates its effectiveness in capturing temporal patterns and modeling complex long-term dependencies within sequential data.

**Figure 2:**
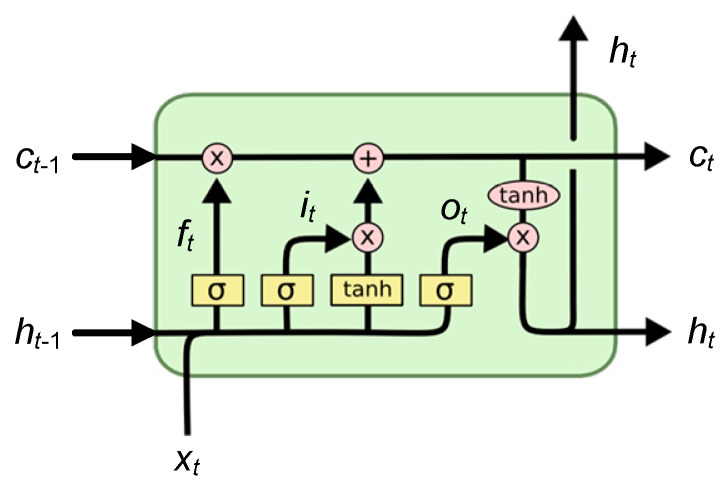
LSTM unit structure.

Figure 2 presents how the LSTM unit transitions its state at step *t*, relying on *x_t_*, *h_t_*_−1_, and *c_t_*_−1_ through four essential stages [34]:

1. Forget Gate Step—determines which components of the prior cell state are to be eliminated. The forget gate *f_t_* is computed by:

(1) $$f_{t}=\sigma \left(W_{f}\cdot x_{t}+U_{f}\cdot h_{t-1}+b_{f}\right)$$

2. Input Gate Step—controls which fresh information can enter cell memory. The input gate *i_t_* and the input modulation gate ${\hat{c}}_{t}$ are computed using the formula:

(2) $$i_{t}=\sigma \left(W_{i}\cdot x_{t}+U_{i}\cdot h_{t-1}+b_{i}\right)$$

(3) $${\hat{c}}_{t}=tahn\left(W_{c}\cdot x_{t}+U_{c}\cdot h_{t-1}+b_{c}\right)$$

3. State Update Step—updates the current state based on the information from the input gate and the forget gate. The memory gate *c_t_* is computed by:

(4) $$c_{t}=f_{t}⊙c_{t-1}+i_{t}⊙{\hat{c}}_{t}$$

4. Output Gate Step—determines the output information *o_t_* from the current cell state. Subsequently, based on the updated cell state *c_t_*, the hidden state *h_t_* is computed.

(5) $$o_{t}=\sigma \left(W_{o}\cdot x_{t}+U_{o}\cdot h_{t-1}+b_{o}\right)$$

(6) $$h_{t}=o_{t}⊙tahn\left(c_{t}\right)$$

where {*W*, *U*, *b*} denote input weights, hidden state weights, and bias terms, respectively; *σ* represents the sigmoid activation function, and *tanh* represents the hyperbolic tangent activation function.

LSTM excels at time-series prediction tasks owing to its ability to process sequential data while preserving long-term dependencies. Accurate time-series prediction heavily relies on previous observations. Through its gating mechanism (forget/input/output gates), LSTM efficiently manages information flow, either preserving relevant information in the current state or discarding obsolete or irrelevant information. This intelligent memory management not only prevents the overfitting problem in extended sequences but also focuses attention on meaningful patterns, proving especially valuable when modeling data with prolonged temporal dependencies or cyclical seasonal behavior [34].

B. FFT-Based Time Series Preprocessing

Consider a function space *X* and its transformed counterpart *Ψ*. Suppose $f\left(t\right)\in X$ is a function in the original space, while F(ω)∈Ψ represents its counterpart in the transformed space.

The function *f*: *R*→*X* maps from the time domain *R* to space *X*, with its argument *t* (in seconds) representing the temporal variable. Correspondingly, the function *F*: *Z*→*Ψ* operates from the complex domain *Z* to space *Ψ*, taking frequency *ω* (in rad/s) as its argument. A functional transformation *φ* mapping from space *X* to *Ψ* is hereby introduced as follows:

(7) φ:X→Ψ

The discrete one-dimensional Fourier transform is a specific instance of the transformation form (7), designed for analyzing sampled data sequences within the temporal domain *X*. Like its continuous version, the discrete Fourier transform maps signals between the temporal domain *X* and the spectral domain *Z*. A distinctive characteristic of this transform lies in its application to discrete data sequences, including both finite and infinite time series. For a finite numerical sequence with *N* samples, the discrete Fourier transform is mathematically expressed as follows:

(8) $$X_{k}=\sum_{n=0}^{N-1}x_{n}e^{-ikn\frac{2\pi }{N}},k\in [0,\;N-1]$$

*x*: a discrete sequence supplied as input to the transform.
*X_k_*: the spectral coefficients obtained through the discrete Fourier transform.
*N*: the total count of samples spanning the discrete sequence.

The spectral parameter collection {*X_k_*|*k* = 0, …, *N* − 1} characterizes the frequency composition of the signal. These parameters reside in the domain of complex numbers *Z*, a space defined by the real and imaginary axes. For any given frequency component *X_k_* within this set, the following relation applies:

(9) $$X_{k}=Re\left(X_{k}\right)\pm iIm\left(X_{k}\right),\;k\in [0,\;N-1]$$

For efficient transmission and processing of spectral parameters within neural network architectures, their polar coordinate representation is employed:

(10) $$X_{k}=\left|X_{k}\right|\left(cos\left(arg\left(X_{k}\right)\right)+isin\left(arg\left(X_{k}\right)\right)\right)$$

where

(11) $$\left|X_{k}\right|=\sqrt{Re(X_{k}{)}^{2}+Im(X_{k}{)}^{2}}$$

(12) $$arg\left(X_{k}\right)=arctan\left(\frac{Im\left(X_{k}\right)}{Re\left(X_{k}\right)}\right)$$

Consequently, Formula (9) is replaced with Formulas (10)–(12), wherein each spectral parameter is uniquely characterized by a pair of quantities derived from the complex number’s polar representation.

Formula (11) determines the modulus (complex number magnitude), representing the spectral parameter’s amplitude. Formula (12) computes the argument (complex number angle), indicating the spectral parameter’s phase. These polar coordinates provide clearer frequency characterization, as they explicitly define the signal’s amplitude and phase components. Magnitude and phase values derived from the frequency components are utilized as features for subsequent machine learning-based time series analysis.

C. Enhanced FFT–LSTM Architecture

LSTM effectively addresses long-term dependency challenges through its memory cell architecture, making it well-suited for classifying activities characterized by distinct temporal patterns [35]. Consequently, the HAR module proposed in this work leverages LSTM to recognize human activities using accelerometer and gyroscope data acquired from a smart device.

**Figure 3:**
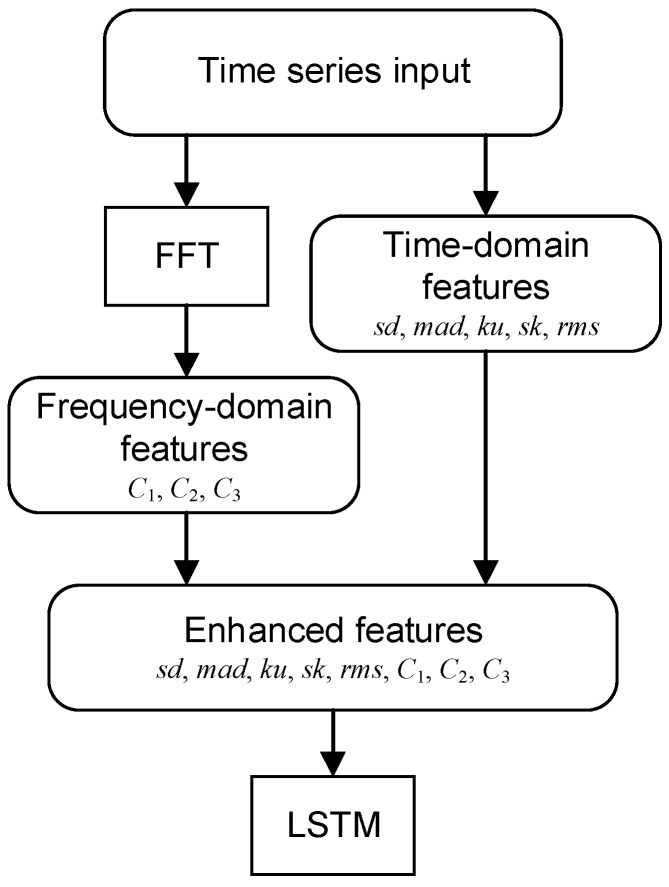
Enhanced FFT–LSTM model.

Time-series signals are frequently corrupted by sensor noise, data gaps, and irregular artifacts, all of which can degrade the predictive capability of an LSTM [36,37]. Consequently, careful preprocessing of raw data is essential for ensuring robust and accurate recognition outcomes. A study by [38] introduced an enhanced LSTM variant that integrates an FFT-based preprocessing stage. Through extensive comparative experiments, the authors validated the efficacy of this FFT–LSTM fusion. Subsequent investigations [39,40] have further examined FFT–LSTM architectures across diverse application domains. Nevertheless, these prior approaches depend solely on frequency-domain features derived via FFT, a constraint that may limit recognition accuracy when applied to the complex, multimodal time series data examined in this study. To address this limitation, this study proposes an enhanced FFT–LSTM architecture that jointly exploits both temporal and spectral representations, as illustrated in Figure 3.

In the proposed architecture, time-domain features and frequency-domain characteristics derived via FFT are jointly supplied to the network, enabling it to simultaneously leverage information from both domains. Consequently, the model gains a richer input representation that encapsulates both temporal and spectral properties.

The following time-domain statistical metrics are employed for feature extraction:

(13) $$\begin{array}{c} sd=\sqrt{\frac{1}{n}\sum_{i=1}^{n}{\left(a_{i}-\bar{a}\right)}^{2}}\\ mad=\frac{1}{n}\sum_{i=1}^{n}\left|a_{i}-\bar{a}\right|\\ ku=\frac{\sum_{i=1}^{n}{\left(a_{i}-\bar{a}\right)}^{4}}{\left(n-1\right)sd^{4}}\\ sk=\frac{\sum_{i=1}^{n}{\left(a_{i}-\bar{a}\right)}^{3}}{\left(n-1\right)sd^{3}}\\ rms=\sqrt{\frac{\sum_{i=1}^{n}a_{i}^{2}}{n}} \end{array}$$

where *sd*, *mad*, *ku*, *sk*, and *rms* represent the standard deviation, mean absolute deviation, kurtosis, skewness, and root mean square, respectively; *a_i_* denotes the *i*-th data point within the window, $\bar{a}$ denotes the mean value of all data points in that window.

The frequency-domain features comprise the three highest peaks of cepstrum coefficients, denoted as *C*_1_, *C*_2_, and *C*_3_. The calculation formula is as follows:

(14) $$C\left(n\right)=\frac{1}{N}\sum_{k=0}^{N-1}\log{\left|X_{k}\right|}^{2}\cdot e^{j2\pi kn/N},\;n=0,1,\ldots ,N-1$$

Let *x_t_* = [*sd*, *mad*, *ku*, *sk*] denote the time-domain feature vector, $\varphi_{t}$ = [*C*_1_, *C*_2_, *C*_3_] represent the frequency-domain feature vector. The operational pipeline of the enhanced FFT–LSTM architecture proceeds as outlined below.

In the forget gate computation, both magnitude and phase components are explicitly incorporated:

(15) $$f_{t}=\sigma \left(W_{f}\cdot \left[x_{t},\varphi_{t}\right]+U_{f}\cdot h_{t-1}+b_{f}\right)$$

The update steps of cell state are as follows:

(16) $$i_{t}=\sigma \left(W_{i}\cdot \left[x_{t},\varphi_{t}\right]+U_{i}\cdot h_{t-1}+b_{i}\right)$$

(17) $${\hat{c}}_{t}=tahn\left(W_{c}\cdot \left[x_{t},\varphi_{t}\right]+U_{c}\cdot h_{t-1}+b_{c}\right)$$

The magnitude and phase components extracted from the time series are subsequently forwarded to the output stage of the network:

(18) $$o_{t}=\sigma \left(W_{o}\cdot \left[x_{t},\varphi_{t}\right]+U_{o}\cdot h_{t-1}+b_{o}\right)$$

At each step, it can be seen that the proposed model will enhance the neural network’s input by incorporating frequency-domain features while preserving the original temporal characteristics. This approach will help to improve recognition reliability across extended time spans for sequential data.

#### 3.2.2. HAR Using the Proposed FFT–LSTM Model

The model recognizes human activities (i.e., the activities of arms or legs) using accelerometer and gyroscope data from wearable devices such as smartphones or smartwatches. The accelerometer and gyroscope respectively capture tri-axial acceleration and angular velocity along the x, y, and z axes. Following [6], the model processes acceleration values with absolute orientation derived from sensor fusion of accelerations and angular velocities via Mahony’s AHRS algorithm [41]. This absolute orientation, represented as a quaternion relative to the device’s local coordinate system, is computed through the AHRS process. By integrating inertial sensor data, the AHRS process improves LSTM-based action recognition by providing robust orientation estimates while eliminating raw sensor noise and drift.

Sliding window sampling is applied to the output of the AHRS process. The continuous time-series stream is partitioned into fixed-length windows of 2.56 s using this technique, with a 50% overlap maintained between adjacent segments. Each window has 128 readings, resulting in a time step of 128.

**Figure 4:**
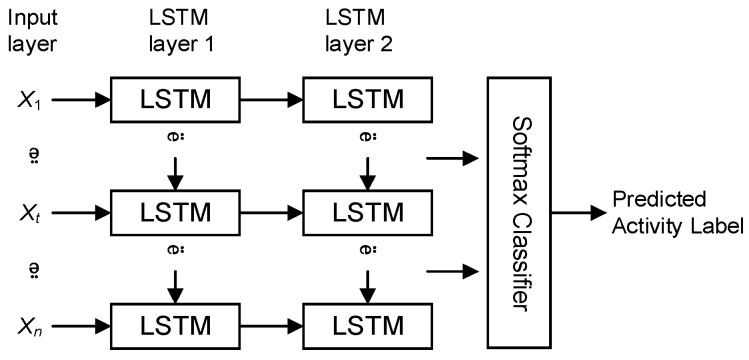
Architecture of the LSTM.

The LSTM module used in the model consists of two stacked layers, which are more effective in representing complex sequential relationships than basic single-Layer LSTMs. Figure 4 illustrates the LSTM architecture. The network extracts and transforms the input into new feature representations, which are subsequently passed to a SoftMax classifier that assigns each feature vector to its corresponding activity category.

The LSTM is optimized using a cross-entropy loss augmented with an L2 regularization term, formulated as follows:

(19) $$loss=-\frac{1}{K}\sum_{i=1}^{K}\left\{y_{i}\log\left(\;{\hat{y}}_{i}\right)\right\}+\lambda \sum_{j=1}^{M}\frac{\omega_{j}^{2}}{2}$$

where *K* denotes the number of activity classes; *y_i_* and ${\hat{y}}_{i}$ correspond to the ground-truth and predicted labels for the *i*-th sample, respectively; *λ* represents the L2 regularization coefficient; *M* indicates the total count of trainable weights within the LSTM; and $\omega_{j}$ refers to the *j*-th individual weight parameter. An adaptive moment estimation (ADAM) optimizer was used for network optimization. The hyperparameter configurations for the LSTM are summarized in Table 1.

### 3.3. Bio-Inspired Adaptive Multimodal Decision Fusion (BA-MDF) Module

**Figure 5:**
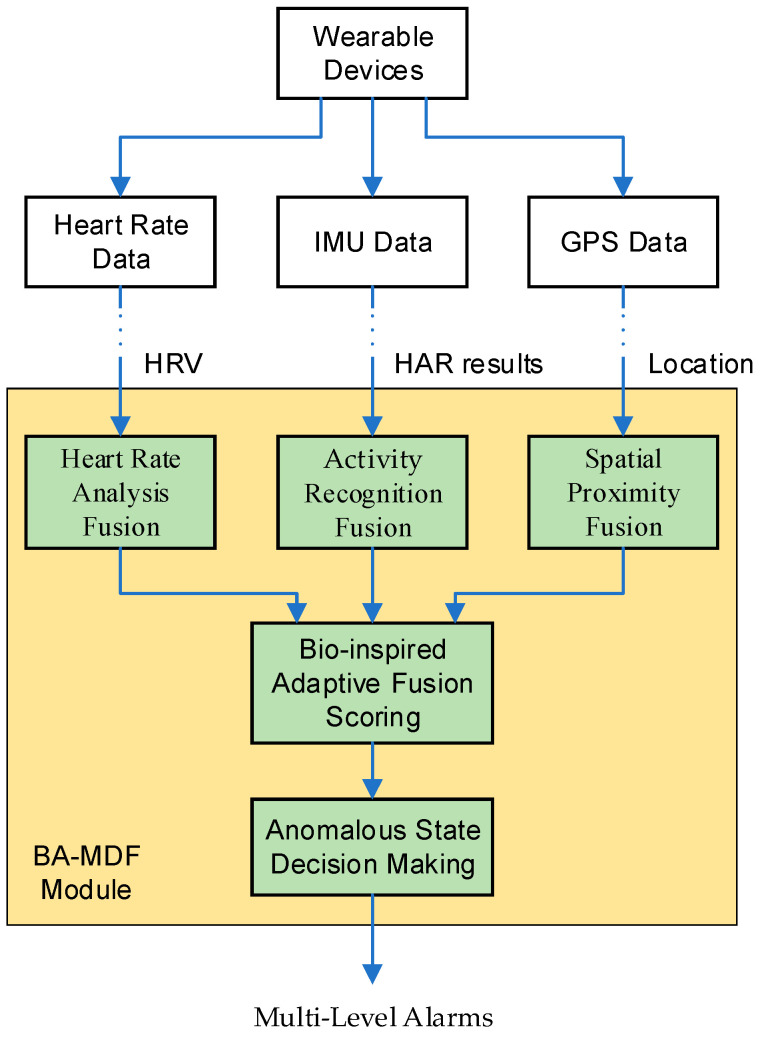
Bio-inspired adaptive fusion and decision-making mechanism.

The core of the multimodal safety monitoring framework is its bio-inspired adaptive fusion and decision-making mechanism, which integrates HAR results, heart rate, and location data to accurately detect hazardous worker states. As illustrated in Figure 5, this mechanism is implemented within the BA-MDF module. Activity recognition, heart rate monitoring, and spatial proximity analysis operate as independent modalities with separate thresholds in a temporal window. Their outputs are unified via a weighted fusion mechanism that combines module-specific scores into a single safety assessment.

The BA-MDF module implements a bio-inspired adaptive fusion mechanism based on the inverse effectiveness principle observed in biological multisensory systems. Unlike conventional fixed-weight fusion, the system dynamically adjusts modality weights based on real-time reliability estimates—mirroring how the superior colliculus enhances weak sensory inputs through cross-modal integration.

A. Activity Recognition Fusion

As described in Section 3.2, the activity recognition module leverages an LSTM-based neural network to classify worker activities from IMU data. A 20 s time window is used to monitor activity duration, enabling the detection of prolonged lying (≥20 s) or simultaneous lying by multiple workers. The activity score (*Score_act_*) is calculated as follows:

(20) $$Score_{act}=\left\{\begin{array}{cc} 0.1 & \;\mathrm{if}\;\mathrm{Normal}\;\mathrm{activity}\\ 1.0 & \;\mathrm{if}\;\mathrm{Prolonged}\;\mathrm{lying} \end{array}\right.$$

B. Heart Rate Analysis Fusion

Heart rate data is analyzed using HRV metrics, calculated as |*H_current_* − *H_avg_*|/*H_avg_*. A threshold (*θ_heart_* = 0.3) identifies significant heart rate fluctuations indicative of physiological stress or distress. The 20 s time window captures temporal heart rate patterns, allowing the system to detect prolonged anomalies (≥10 s) or severe anomalies. The heart rate score (*Score_heart_*) is assigned as follows:

(21) $$Score_{heart}=\left\{\begin{array}{cc} 0.05 & \;\mathrm{if}\;\mathrm{Normal}\;\mathrm{heart}\;\mathrm{rate}\\ 0.7 & \;\mathrm{if}\;\mathrm{prolonged}\;\mathrm{anomalies}\\ 0.9 & \;\mathrm{if}\;\mathrm{severe}\;\mathrm{anomaly} \end{array}\right.$$

This scoring system prioritizes prolonged and severe anomalies, ensuring that physiological stress is promptly detected.

C. Spatial Proximity Fusion

Spatial proximity is analyzed using GPS coordinates and the Haversine formula to compute geographic distances between workers. A threshold (*D_threshold_* = 5 m) defines spatial adjacency. If a worker is in proximity to another anomalous worker, their activity score is weighted by 1.2 (*Score_group_* = *Score_act_* × 1.2). This integration enhances the detection of group-related hazards, such as multiple workers simultaneously lying in close proximity. In situations where GPS is not available, such as in a closed environment, UWB positioning technology can be used as a substitute.

D. Bio-inspired Adaptive Fusion Scoring

The system employs a weighted scoring approach to combine activity, physiological, and spatial data:

(22) $$\begin{array}{c} Score_{total}=w_{1}(t)\times Score_{act}+w_{2}(t)\times Score_{heart}\\ +w_{3}(t)\times Score_{group} \end{array}$$

For the *i*-th modality at time *t*, its inverse effective weight $w_{i}(t)$ is calculated as:

(23) $$w_{i}(t)=\frac{R_{i}(t)}{\sum_{j}R_{j}(t)}$$

where $R_{i}(t)\in [0,1]$ denotes the real-time reliability of modality *i*. Following [42], the reliability is computed as the inverse of the normalized signal variance, $R_{i}\left(t\right)=1/\left(\sigma_{norm}^{2}+\epsilon \right)$, where ϵ is a small positive constant added for numerical stability. When a modality’s reliability decreases due to environmental interference, its weight automatically decreases, compelling the system to rely on alternative reliable modalities. Consequently, the benefits of multimodal integration are actually enhanced. This adaptive mechanism ensures robust fusion under sensor degradation without requiring manual threshold tuning.

E. Decision Mechanism

The decision mechanism triggers alarms based on the total score:

(24) $$S=\left\{\begin{array}{cc} 0\;(\mathrm{Normal}\;\mathrm{state}) & \mathrm{if}\;Score_{total}\leq 0.3\\ 1\;(\mathrm{Level}\;1\;\mathrm{alarm}) & \mathrm{if}\;0.3<Score_{total}\leq 0.6\\ 2\;(\mathrm{Level}\;2\;\mathrm{alarm}) & \mathrm{if}\;Score_{total}>0.6 \end{array}\right.$$

Normal state only needs routine monitoring; Level 1 alarm suggests moderate risks that need monitoring; and Level 2 alarm indicates serious hazards requiring immediate attention. This multi-level alarm system ensures that the most critical hazards are prioritized while maintaining sensitivity to moderate risks.

The multimodal safety monitoring process, which integrates the HAR and BA-MDF modules, is formalized in Algorithm 1.


**Algorithm 1** Multimodal Safety Monitoring**Input:** IMU sensor data *D*, Heart rate data *H*, GPS coordinates *C***Output:** Anomalous state flag *S*1. Initialize parameters:      - Time window *T* = 20 s      - Activity history record *Hist_act_* = []      - Heart rate history record *Hist_heart_* = []2. Process IMU sensor data *D*:      - Preprocess IMU sensor data *D*      - Predict activity *P* using the enhanced FFT-LSTM model      - If *P* is lying        * Record lying start time *t_start_*        * Monitor lying duration *Dur* = *t_current_* − *t_start_*      - Update *Hist_act_*3. Analyze heart rate data *H*:      - Get current heart rate *H_current_*      - Calculate *HRV* = | *H_current_* − *H_avg_*|/*H_avg_*      - If *HRV* > *θ_heart_*, mark as heart rate anomaly      - Update *Hist_heart_*4. Detect GPS proximity:      - Get current GPS coordinates *C*      - Calculate distance *D* to nearby workers using Haversine formula      - If *D* ≤ *D_threshold_*, mark as nearby worker5. Bio-inspired adaptive fusion scoring using Equation (22)6. Anomalous state decision making using Equation (24), and return *S*


Unlike classic adaptive fusion techniques that often require pre-defined thresholds or heuristic rules for weight adjustment [27,28], the proposed BA-MDF formalizes weight adaptation through a continuous, uncertainty-driven mechanism (Equation (23)) inspired by biological sensory integration. This provides a principled mathematical framework for weight dynamics. For instance, in contrast to static weighted averaging or switching logic, our model offers a smooth, gradient-like transition of influence based on real-time uncertainty estimates.

The BA-MDF method is distinguished from standard reliability-weighted fusion by three principal differences. First, its weight adaptation rule is directly derived from the neuroscientific inverse effectiveness principle, providing a biological justification for the fusion strategy rather than an empirical or optimization-based one. Second, weights are continuously adjusted based on real-time uncertainty estimates, not just triggered by binary sensor failure flags. Third, it is specifically designed to maintain performance under partial sensory degradation (simulating conditions like smoke, dust, or signal loss common in confined spaces), a scenario where conventional fusion often fails catastrophically, as validated in our experiments. This makes BA-MDF not merely a heuristic rule-set but a formally modeled, bio-inspired adaptive fusion framework for safety-critical environments.

## 4. Results and Discussion

### 4.1. Performance Evaluation of HAR

#### 4.1.1. Dataset Description and Preprocessing

The HAR experiments using the enhanced FFT–LSTM model were conducted on Reyes’s dataset [43], which covers six activity categories: walking, ascending stairs, descending stairs, sitting, standing, and lying. Measurements were obtained from accelerometer and gyroscope sensors embedded in smartphones secured at the waist of participating volunteers. All signals were sampled at a frequency of 50 Hz. To address overfitting problems, as in [35], the dataset was randomly divided into two subsets for the LSTM process, with 70% for training and the remaining 30% for evaluation.

**Figure 6:**
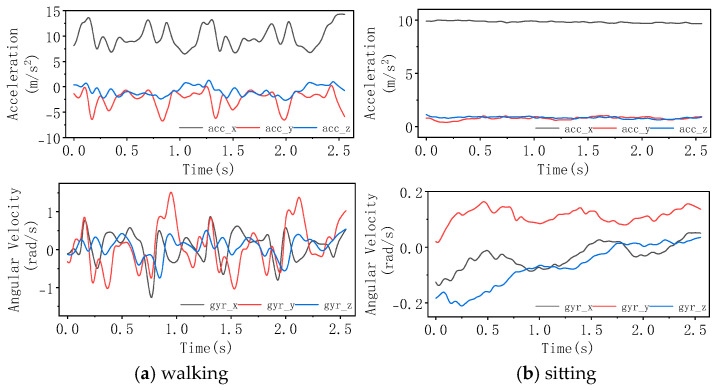
Sample accelerometer and gyroscope data.

**Figure 7:**
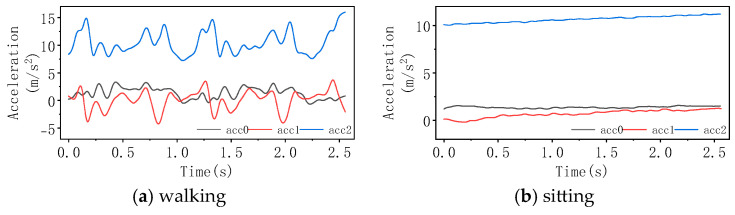
AHRS results for sample data in Figure 6.

Some sample data in the dataset are shown in Figure 6. For the original data shown in Figure 6, Figure 7 depicts the result of the AHRS process.

#### 4.1.2. Comparative Analysis

The performance of the enhanced FFT–LSTM model for HAR was benchmarked against several established approaches. The primary baseline is a classical time-domain LSTM architecture [44], which processes accelerometer signals derived from the AHRS preprocessing pipeline. The second baseline is the FFT–LSTM model proposed in [38], which operates entirely in the frequency domain. This approach transforms time-series signals by substituting the original temporal sequence with a compact frequency-domain representation. Specifically, it utilizes the FFT results computed from the AHRS-processed accelerometer signals as input to the LSTM. Our approach leverages both time-domain and frequency-domain representations, combining the AHRS-processed accelerometer signals with their corresponding FFT-derived spectral features.

**Figure 8:**
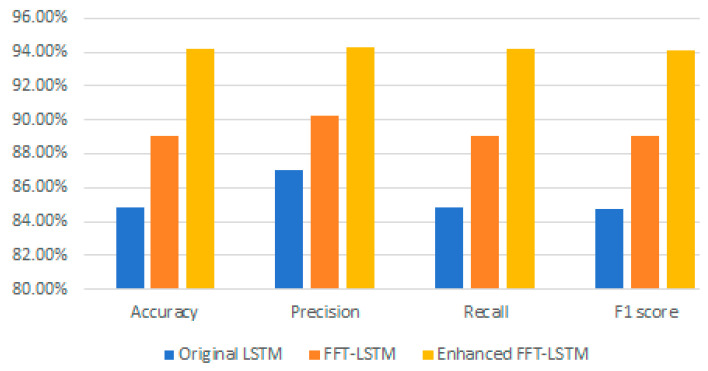
Performance of different versions of the LSTM.

Figure 8 presents a comparative summary of the performance across different LSTM variants. The FFT–LSTM model outperforms the original LSTM by approximately 5% across all four evaluation metrics—accuracy, precision, recall, and F1 score. Furthermore, the enhanced FFT–LSTM achieves an even more substantial improvement, surpassing the original LSTM by roughly 11% on the same metrics.

**Figure 9:**
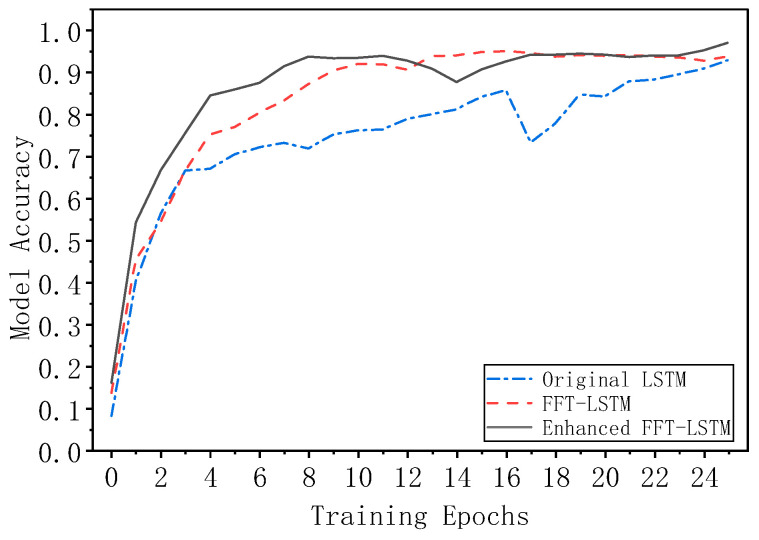
Training accuracy of different versions of LSTM over training epochs.

The training time also varies with the version of LSTMs. Figure 9 illustrates the accuracy trajectories of different LSTM variants across training epochs. The original LSTM attains 90% accuracy by the 21st epoch and further climbs to 95% at the 24th epoch. In contrast, the FFT–LSTM reaches 90% accuracy considerably earlier, at the 8th epoch, and achieves 95% by the 13th epoch. The enhanced FFT–LSTM reaches 90% accuracy at the 10th epoch and further improves to 95% by the 13th epoch. Consequently, both the FFT–LSTM and the enhanced FFT–LSTM reduce training time by approximately 50% relative to the original LSTM.

Generally, the enhanced FFT–LSTM demonstrates dual improvements in HAR performance and computational efficiency, as validated by its superior accuracy/F1 scores (Figure 8) and a 50% reduction in training time compared to baseline methods (Figure 9).

The confusion matrices in Table 2 demonstrate the classification performance using different versions of LSTM. As can be seen from the confusion matrices, the proposed approach attains superior performance across all six activity categories, although recognition accuracy varies substantially among individual activities. Lying achieves the highest recognition rate in the matrices, possibly due to its distinct triaxial acceleration and angular velocity patterns compared to other activities. Sitting and Standing exhibit a modest degree of mutual confusion within the matrices, though the overall misclassification rate between them remains relatively low.

#### 4.1.3. Impact of Hidden Unit Count on Model Performance

**Figure 10:**
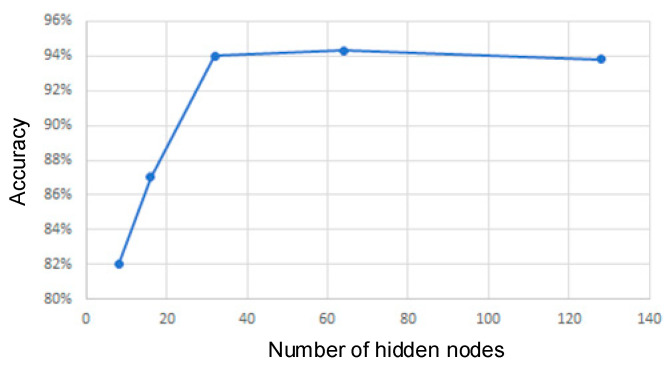
Recognition accuracies with different hidden counts.

To examine the influence of the hidden node count—a critical hyperparameter in the proposed FFT–LSTM architecture—on HAR performance, a dedicated experiment was conducted. The corresponding results are presented in Figure 10.

With only eight hidden nodes, the model yields a modest accuracy of 82%. Expanding the hidden node count from 8 to 64 drives accuracy above 94%. Beyond this point, further increments produce negligible gains. Balancing predictive performance against computational cost—since additional hidden nodes prolong training duration—a configuration of 32 hidden nodes is adopted as optimal for the proposed FFT–LSTM model for HAR tasks.

### 4.2. System Experiments

The system was validated under controlled industrial conditions to detect prolonged supine postures (≥20 s) and co-occurring multi-worker hazards. Performance was assessed using data from simulated enclosed workspaces, with detection accuracy, false alarm rate, and temporal response latency as key metrics. All experiments were conducted on a Nuvo-6108GC embedded industrial computer (Intel^®^ Core™ i7-6700TE, NVIDIA^®^ GeForce GTX 1050Ti GPU, 32GB RAM) running Python 3.8, which was sourced from Neousys Technology Inc., New Taipei City, China.

#### 4.2.1. Experimental Design and Data Collection

**Figure 11:**
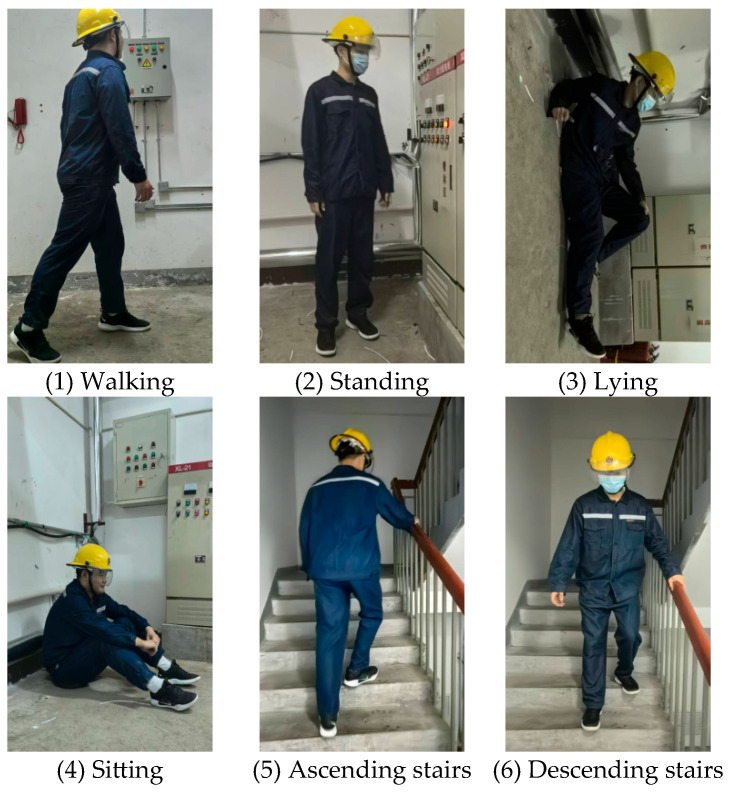
Typical behaviors in real-world scenarios.

A controlled test environment was established at a power construction site. Wearable IMUs were deployed to capture six categories of typical worker behaviors (Figure 11) from 12 construction personnel (aged 25–45) under real-world working conditions:

- Walking (average velocity: 0.8–1.2 m/s)
- Standing (including static working postures)
- Lying (simulating accidental falls)
- Sitting (resting state)
- Ascending stairs (30-degree incline)
- Descending stairs (30-degree incline)

Data sampling was configured at 50 Hz over a 6 h continuous acquisition period, yielding 1550 validated data samples.

Dual-triggering criteria were set for the safety warning system:

- Single-person lying state ≥ 20 s (medically confirmed threshold for unconsciousness)
- More than two simultaneous lying postures in the detection zone (preventing mass incidents)

#### 4.2.2. Experimental Results and Discussion

A. Field Validation of the Model Performance

The enhanced FFT–LSTM model developed in this study was applied to the collected data for behavior recognition, with five-fold cross-validation revealing an overall accuracy of 92.4% (±1.7%), the highest recognition accuracy for lying postures (96.2%), and a misjudgment rate of 7.3% for stair motions, primarily occurring under tool-carrying conditions.

B. Field Validation of Anomaly Detection

During on-site accident simulation at the construction site, the system successfully identified all pre-configured emergency scenarios, including five individual unconsciousness events and two simulated group fall incidents. The alarm response latency was maintained under 800 ms, while the average rescue arrival time following triggered alerts was reduced to 43 s. Notably, the proposed multimodal monitoring system remains fully operational in zero-light or smoke-filled confined spaces where conventional video surveillance is inherently non-functional.

The adaptive fusion mechanism demonstrated particular robustness under simulated sensor degradation. When GPS signals were artificially attenuated (underground scenario), the system automatically increased IMU and heart rate weights, maintaining 94.3% (±1.8%) detection accuracy versus 78.2% (±3.1%) for fixed-weight fusion—validating the bio-inspired inverse effectiveness design. The results are reported with 95% confidence intervals and standard deviations across five runs.

To further validate the advantage of adaptive fusion, the full BA-MDF pipeline was compared against three alternative fusion strategies under identical conditions. As summarized in Table 3, BA-MDF achieved 94.3% accuracy, substantially outperforming fixed-weight fusion (78.2%), majority voting (81.6%), and Bayesian fusion (85.9%). This 8.4–16.1% improvement confirms that the bio-inspired adaptive weighting mechanism provides a decisive advantage over both static and classical probabilistic fusion approaches, particularly under partial sensor degradation.

Several limitations of the field validation should be noted: all findings derive from controlled simulations rather than real-world deployment, and the modest dataset (12 participants) warrants broader validation. Expanded real-world testing is planned.

## 5. Conclusions

To ensure operational safety in confined spaces, this study proposes an intelligent anomalous situation detection method based on multimodal data analysis. The proposed safety monitoring framework consists of two core components: the human activity recognition (HAR) module and the bio-inspired adaptive multimodal decision fusion (BA-MDF) module.

The BA-MDF module collects and processes multimodal sensor data—including accelerometer, gyroscope, heart rate, and GPS signals—from wearable devices like smartphones or smartwatches to monitor human activities. HAR serves as the fundamental technology for human activity monitoring. To improve HAR performance, an enhanced FFT–LSTM architecture was developed that integrates both time-domain and frequency-domain features. Experimental results for HAR demonstrate that this model significantly outperforms baseline methods in terms of accuracy, precision, recall, and F1-score metrics.

The BA-MDF module integrates HAR outputs, HRV trends, and geospatial proximity constraints into a deterministic rule-based scoring system. It dynamically evaluates risk by mapping activity states (e.g., prolonged lying, elevated HRV) against context-aware safety thresholds and triggers tiered alerts via an adaptive fusion mechanism.

The system demonstrated a 92.4% overall accuracy in real-world HAR testing. During simulated accident scenarios, the system achieved reliable identification accuracy for all pre-defined emergency cases (including unconsciousness and fall events) with inference latency below 800 ms. The average rescue arrival time following triggered alerts was reduced to 43 s. Critically, the proposed multimodal monitoring system remains fully operational in environments where conventional video surveillance is inherently non-functional. The experimental results demonstrate that the proposed method can effectively reduce risks from anomalous events in visually restricted confined spaces, where conventional surveillance systems face operational constraints, thereby enhancing overall safety management in such challenging environments.

Beyond technical contributions, this work demonstrates the value of bio-inspired design principles in industrial AI systems. The inverse effectiveness mechanism—borrowed from neuroscience—provides a theoretically grounded solution to sensor degradation that would be difficult to discover through purely empirical optimization. Future work will explore additional biological principles, such as spike-based efficient coding and neuromodulatory gain control, for next-generation safety monitoring systems.

The thresholds (e.g., *θ_heart_* = 0.3, *D_threshold_* = 5 m) and scoring values (e.g., 0.1, 0.7, 1.0) used in the BA-MDF module were determined empirically during system development to demonstrate the framework’s feasibility. While effective in the controlled experiments, these parameters may benefit from further theoretical optimization or data-driven calibration to improve generalizability across diverse environments. Future work will focus on establishing a more rigorous theoretical basis for these parameters, potentially through probabilistic modeling or machine learning techniques, to enhance the system’s adaptability and performance.

## Figures and Tables

**Table 1:** LSTM Configuration Specifications.

Parameter Name	Value
Time step	128
Training epochs	300
Learning rate	0.0025
Hidden layer count	2
Hidden unit dimensionality	32
Maximum iterations	1000
Mini-batch size	1500
Lambda for L2 regularization	0.0015
Loss rate	0.0015

**Table 2:** Confusion matrices using different versions of LSTM.

(a) Original LSTM							
		Predicted					
		Walking	Ascending Stairs	Descending Stairs	Sitting	Standing	Lying
Actual	Walking	0.872	0.052	0.076	0.0	0.0	0.0
	Ascending stairs	0.074	0.863	0.063	0.0	0.0	0.0
	Descending stairs	0.057	0.079	0.864	0.0	0.0	0.0
	Sitting	0.0	0.012	0.0	0.768	0.22	0.0
	Standing	0.062	0.0	0.0	0.057	0.881	0.0
	Lying	0.0	0.0	0.0	0.04	0.077	0.883
(b) FFT–LSTM							
		Predicted					
		Walking	Ascending Stairs	Descending Stairs	Sitting	Standing	Lying
Actual	Walking	0.912	0.032	0.056	0.0	0.0	0.0
	Ascending stairs	0.049	0.913	0.038	0.0	0.0	0.0
	Descending stairs	0.027	0.049	0.924	0.0	0.0	0.0
	Sitting	0.0	0.01	0.0	0.808	0.182	0.0
	Standing	0.032	0.0	0.0	0.047	0.921	0.0
	Lying	0.0	0.0	0.0	0.0	0.067	0.923
(c) Enhanced FFT–LSTM							
		Predicted					
		Walking	Ascending Stairs	Descending Stairs	Sitting	Standing	Lying
Actual	Walking	0.962	0.007	0.031	0.0	0.0	0.0
	Ascending stairs	0.034	0.943	0.023	0.0	0.0	0.0
	Descending stairs	0.007	0.029	0.964	0.0	0.0	0.0
	Sitting	0.0	0.006	0.0	0.874	0.12	0.0
	Standing	0.012	0.0	0.0	0.027	0.961	0.0
	Lying	0.0	0.0	0.0	0.0	0.037	0.963

**Table 3:** Full-pipeline fusion strategy comparison.

Fusion Strategy	Accuracy (%)	Precision (%)	Recall (%)	F1-Score (%)
Fixed-Weight Fusion	78.2	75.4	72.8	74.1
Majority Voting	81.6	79.1	76.3	77.7
Bayesian Fusion	85.9	83.2	82.5	82.8
BA-MDF	94.3	93.1	92.7	92.9

## Data Availability

The original contributions presented in this study are included in the article. The full source code is not publicly available due to its integration into a broader proprietary software ecosystem but may be made available from the corresponding author upon reasonable request.
